# Use of gastric acid–suppressive agents increases the risk of dementia in patients with upper gastrointestinal disease: A population-based retrospective cohort study

**DOI:** 10.1371/journal.pone.0249050

**Published:** 2021-03-25

**Authors:** Hsiu-Chen Lin, Kuan-Tzu Huang, Hsiu-Li Lin, Yow-Sheng Uang, Yi Ho, Joseph Jordan Keller, Li-Hsuan Wang

**Affiliations:** 1 Department of Pediatrics, School of Medicine, College of Medicine, Taipei Medical University, Taipei, Taiwan; 2 Department of Clinical Pathology, Taipei Medical University Hospital, Taipei, Taiwan; 3 School of Pharmacy, College of Pharmacy, Taipei Medical University, Taipei, Taiwan; 4 Division of Clinical Pharmacy, Department of Pharmacy, Taipei Veterans General Hospital, Taipei, Taiwan; 5 Department of Neurology, General Cathay Hospital, New Taipei City, Taiwan; 6 College of Medicine, Ohio State University, Columbus, Ohio, United States of America; 7 Department of Pharmacy, Taipei Medical University Hospital, Taipei, Taiwan; Istituto Di Ricerche Farmacologiche Mario Negri, ITALY

## Abstract

**Background:**

Prescriptions for gastric acid–suppressive agents, including proton-pump inhibitors (PPIs) and histamine type-2 receptor antagonists (H2RAs), are rising. However, little data exist regarding their association with dementia in the Asian population. The objective of this study was thus to investigate the impact of the use of PPIs and H2RAs on the risk of dementia in an Asian population with upper gastrointestinal disease (UGID).

**Methods:**

We conducted a population-based retrospective cohort study with a 10-year follow-up using data from 2000 to 2015 derived from Taiwan’s Longitudinal Health Insurance Database. We included 6711 patients with UGID receiving gastric acid–suppressive agents, 6711 patients with UGID not receiving agents, and 6711 patients without UGID or treatment thereof, all at least 20 years of age. Groups were matched for age, sex, and index date. The association between gastric acid–suppressive agent use and dementia was analyzed using a Cox proportional hazards regression model adjusted for potential confounders.

**Results:**

The adjusted hazard ratio (aHR) of dementia for patients with UGID receiving gastric acid–suppressive agents compared with patients with UGID without gastric acid–suppressive agents was 1.470 (95% confidence interval [CI] 1.267–1.705, *p* < 0.001). Both PPIs and H2RAs increase the risk of dementia (PPIs: aHR 1.886 [95% CI 1.377–2.582], *p* < 0.001; H2RAs: aHR 1.357 [95% CI 1.098–1.678], *p* < 0.01), with PPIs exhibiting significantly greater risk (aHR 1.456 [95% CI 1.022–2.075], *p* < 0.05).

**Conclusions:**

Our results demonstrate an increased risk of dementia in patients with UGID receiving gastric acid–suppressive agents, including PPIs and H2RAs, and the use of PPIs was associated with a significantly greater risk than H2RA use.

## Introduction

According to the World Health Organization, dementia is a syndrome usually of a chronic or progressive nature caused by a variety of brain illnesses that affect memory, thinking, behavior, and the ability to perform everyday activities. In 2015, the number of people with dementia worldwide was estimated to be 47 million; this number is expected to increase to 75 million in 2030 and 132 million in 2050 [1]. The disease has an enormous socioeconomic cost. In 2010, the estimated worldwide cost of dementia was US$604 billion; it was US$818 billion in 2015 and is set to increase in coming years [1,2]. Hence, factors that potentially increase the risk of dementia must be identified.

The rising number of prescriptions for gastric acid–suppressive agents including proton-pump inhibitors (PPIs) and histamine type-2 receptor antagonist (H2RAs), the standard treatment for upper gastrointestinal disease (UGID), has prompted research regarding whether either medication increases the risk of dementia. One study reported that PPIs are associated with greater risk of dementia in Germans aged 75 and older [3], and another reported an increased risk for dementia among Asian PPI users [4]. A third study determined that H2RA use more than doubles the risk of cognitive impairment in African Americans [5].

Because the previous study of an Asian population investigated only the effect of PPIs on the risk of dementia in the general population, we conducted a retrospective cohort study to investigate the effect of gastric acid–suppressive agents, including both PPIs and H2RAs, on the risk of dementia among an Asian population with UGID.

## Methods

### Data source

Analyses were conducted using a longitudinal sample of patients from Taiwan’s Longitudinal Health Insurance Database (LHID), a national database containing 2 million enrollees randomly selected from the registry of National Health Insurance (NHI) beneficiaries in Taiwan. Data access was provided by the Health and Welfare Data Science Center, Ministry of Health and Welfare, Taiwan. The NHI system covers 99.6% of the national population in Taiwan, totaling approximately 23 million people. The database includes information on age, sex, diagnosis codes (categorized according to International Classification of Diseases, Ninth Revision, Clinical Modification [ICD-9-CM] codes), drug prescriptions (categorized according to Anatomical-Therapeutic Chemical Classification System codes), hospital visits (including detailed clinical and demographic information of all hospital admissions), and ambulatory visits during 2000–2015. This study was based on anonymized administrative claims data and never involved patients directly; the identification numbers of all individuals in the database are encrypted to protect privacy. The study was conducted according to the Declaration of Helsinki and was approved by the Institutional Review Board of Taipei Medical University, Taipei, Taiwan (TMU-JIRB No. 201706019).

### Study patients

This was a retrospective cohort study. We aggregated the data with a 5-year baseline from 2001 to 2005 and followed up by 10-year intervals for each individual. We identified patients newly diagnosed with UGID (ICD-9-CM codes 530–536) between January 1, 2001, and December 31, 2005. Two consecutive diagnoses were required to enhance diagnosis validity. For each patient, we assigned the first date of receiving gastric acid–suppressive agents for treatment of UGID as the index date. We also identified patients without UGID between January 1, 2000, and December 31, 2015, as a comparison group. The records of drug prescriptions were reviewed to determine which individuals had ever received prescriptions for gastric acid–suppressive agents during 2001–2015 after the respective index dates. We classified patients with UGID into two groups: those who received gastric acid–suppressive agents and those who did not. In the group of patients with UGID receiving gastric acid–suppressive agents, we excluded patients who had been diagnosed with dementia before the index date. We also excluded patients with UGID receiving gastric acid–suppressive agents after December 31, 2005, and those receiving gastric acid–suppressive agents for less than 90 days within 365 days after first being administered the agents. Patients with UGID receiving gastric acid–suppressive agents were selected as study group I, and patients with UGID but not receiving gastric acid–suppressive agents were study group II. Each patient in study group I was matched to one in study group II by age, sex, and index year. Patients without UGID were selected as a comparison group. Each patient in study group II was matched to one patient in the comparison group by age, sex, and index year. The outcome of interest was dementia defined as a diagnosis (ICD-9-CM codes 331.0, 331, 290.0, 290.20, 290.21, 290.3). We identified patients with at least two diagnoses in the follow-up period. All individuals were followed for 10 years or censored at the date of dementia diagnosis.

### Medication usage and other relevant information

Study group I received gastric acid–suppressive agents before December 31, 2005. Use of gastric acid-suppressive agents was defined as a prescription for at least one type of PPI (omeprazole, esomeprazole, lansoprazole, dexlansoprazole, pantoprazole, or rabeprazole) or H2RA (cimetidine, ranitidine, famotidine, nizatidine, or roxatidine) for at least 90 days within 365 days after first being administered one of these agents.

#### Covariate ascertainment and adjustment

We adjusted for numerous confounding factors that influence the occurrence of dementia, including age, sex, alcohol abuse, tobacco dependence, comorbidities (diabetes, stroke, hypertension, hyperlipidemia, atherosclerosis, depression, cognitive deficit, head injury, obesity), and drugs (antiplatelet agents, antidiabetes agents, antihypertension agents, antidepressants, statins, NSAIDs, anticholinergic agents, anti-Parkinson agents, antithrombotics, antipsychotics, benzodiazepines, antiarrhythmics, opioid analgesics, steroids).

### Statistical analysis

Statistical analyses were performed using SAS version 9.4. Student’s t test and Pearson’s chi-squared test were applied to evaluate differences in baseline characteristics such as age, sex, comorbidities, and drugs among different groups. Cox proportional hazard ratios (HRs) were used to estimate HRs and 95% confidence intervals (CIs). The Kaplan–Meier method and log-rank test were used to examine the differences in 10-year dementia occurrence rates between the study groups and comparison group. The p values were two-sided, and p < 0.05 was considered statistically significant.

## Results

**Fig 1:**
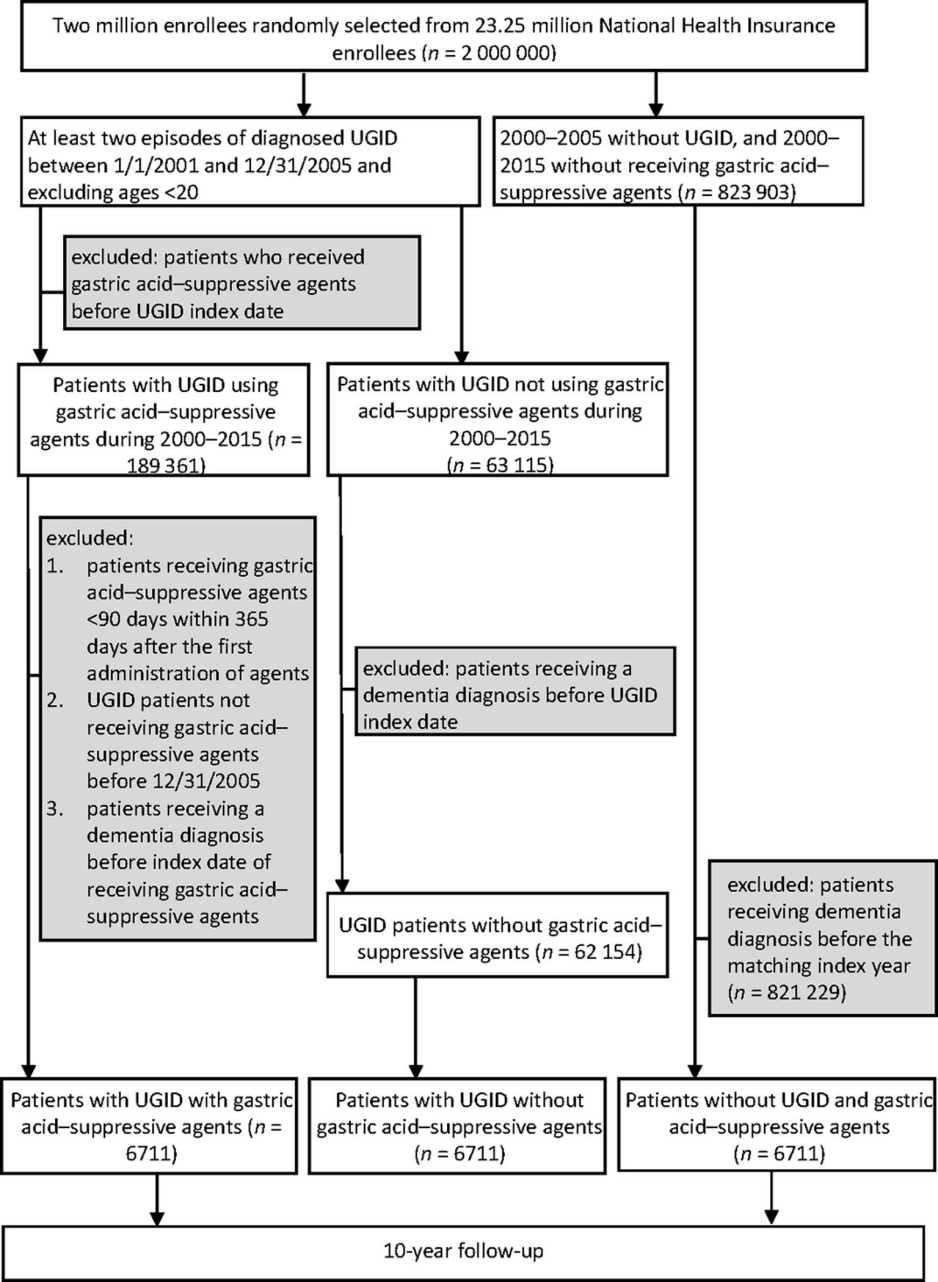
Flowchart of patient inclusion for analysis.

We identified 506 668 patients newly diagnosed with UGID and 823 903 patients without UGID between January 1, 2001, and December 31, 2005. Fig 1 summarizes the enrollment criteria for study group I, study group II, and the comparison group. The latter two groups were 1:1 matched with group I by age, sex, and index year. We identified 6711 patients with UGID receiving gastric acid–suppressive agents as study group I, 6711 patients with UGID but not receiving gastric acid–suppressive agents as study group II, and 6711 patients without UGID as a comparison group.

Table 1 lists the demographic characteristics of patients with and without UGID. After matching, the distributions of age and sex were similar among the three groups. The mean age was 55.02 ± 15.67 years, and 57.06% of the individuals were male. We estimated the crude HRs and adjusted HRs (aHRs) for the risk of dementia between study group I and study II and between study group II and the comparison group. During the 10-year follow-up, the aHR of dementia for study group I compared with study group II was 1.486 (95% CI 1.283–1.721, *p* < 0.001), and the aHR of dementia for study group II compared with the comparison group was 1.129 (95% CI 0.947–1.346, *p* > 0.05; Table 2). After subgroup analysis, both only-PPI and only-H2RA use increased the risk of dementia compared with study group II (only-PPI use: aHR 1.836 [95% CI 1.345–2.507], *p* < 0.001; only-H2RA use: aHR 1.359 [95% CI 1.102–1.676], *p* < 0.01), and use of PPIs was associated with a significantly greater risk than was use of H2RAs (aHR 1.462 [95% CI 1.038–2.057], *p* < 0.05; Table 3).

**Table 1:** Demographic characteristics of patients with and without UGID.

Variables (%)	UGID Patients				
	Study Group I	Study Group II	Comparison Group	*p* value^a^	*p* value^b^
	*n* = 6711	*n* = 6711	*n* = 6711		
Age, years (mean ± SD)	55.02 ± 15.67	55.02 ± 15.67	55.02 ± 15.67	1	1
Gender/Male (n, %)	3829 (57.06%)	3829 (57.06%)	3829 (57.06%)	1	1
Alcohol abuse (n, %)	26 (0.39%)	15 (0.22%)	10 (0.15%)	0.0853	0.3169
Tobacco use disorder (n, %)	21 (0.31%)	18 (0.27%)	5 (0.07%)	0.6305	0.0067
Head injury (n, %)	< 5^f^	9 (0.13%)	< 5^f^	0.0347	0.1653
Obesity (n, %)	44 (0.66%)	41 (0.61%)	17 (0.25%)	0.7441	0.0016
Diabetes (n, %)	1619 (24.12%)	1240 (18.48%)	699 (10.42%)	< 0.0001	< 0.0001
Hypertension (n, %)	3000 (44.70%)	2460 (36.66%)	1508 (22.47%)	< 0.0001	< 0.0001
Depression (n, %)	164 (2.44%)	113 (1.68%)	45 (0.67%)	0.002	< 0.0001
Stroke (n, %)	757 (11.28%)	626 (9.33%)	399 (5.95%)	0.0002	< 0.0001
Hyperlipidemia (n, %)	1692 (25.21%)	1193 (17.78%)	541 (8.06%)	< 0.0001	< 0.0001
Atherosclerosis (n, %)	163 (2.43%)	118 (1.76%)	49 (0.73%)	0.0067	< 0.0001
Cognitive deficits (n, %)	20 (0.30%)	19 (0.28%)	< 5^f^	0.8726	0.0002
Anticholinergic agents (n, %)	5415 (80.69%)	4783 (71.27%)	2707 (40.34%)	< 0.0001	< 0.0001
Anti-Parkinson (n, %)	94 (1.40%)	119 (1.77%)	80 (1.19%)	0.0842	0.0053
Antidiabetes agents (n, %)	1113 (16.58%)	881 (13.13%)	538 (8.02%)	< 0.0001	< 0.0001
Antihypertension agents (n, %)	3715 (55.36%)	2902 (43.24%)	1680 (25.03%)	< 0.0001	< 0.0001
Antithrombotics (n, %)	90 (1.34%)	77 (1.15%)	37 (0.55%)	0.3114	0.0002
Antiplatelet agents (n, %)	319 (4.75%)	178 (2.65%)	85 (1.27%)	< 0.0001	< 0.0001
Antipsychotics (n, %)	93 (1.39%)	77 (1.15%)	57 (0.85%)	0.2168	0.0825
Antidepressants (n, %)	634 (9.45%)	362 (5.39%)	149 (2.22%)	< 0.0001	< 0.0001
Benzodiazepines (n, %)	443 (6.60%)	269 (4.01%)	125 (1.86%)	< 0.0001	< 0.0001
Antiarrhythmics (n, %)	333 (4.96%)	259 (3.86%)	110 (1.64%)	0.0019	< 0.0001
Opioid analgesics (n, %)	7 (0.10%)	< 5^f^	< 5^f^	0.3655	0.7054
Steroids (n, %)	2509 (37.39%)	2080 (30.99%)	1056 (15.74%)	< 0.0001	< 0.0001
NSAIDS (n, %)	806 (12.01%)	663 (9.88%)	265 (3.95%)	< 0.0001	< 0.0001
Statins (n, %)	675 (10.06%)	457 (6.81%)	201 (3.00%)	< 0.0001	< 0.0001

†UGID, upper gastrointestinal disease; SD, standard deviation.

†*p* value ^a^: Study group I (UGID with gastric acid-suppressive agents) vs study group II (UGID without gastric acid–suppressive agents).

† *p* value ^b^: Study group II (UGID without gastric acid–suppressive agents) vs comparison group (without UGID and without gastric acid–suppressive agents).

†f: Under Health and Welfare Data Science Center, Ministry of Health and Welfare regulations, statistical results of less than 3 units cannot be disclosed.

**Table 2:** Risk of dementia between study group I, study group II, and comparison group.

Results	UGID patients		
	Study Group I	Study Group II	Comparison Group
	*n* = 6711	*n* = 6711	*n* = 6711
Dementia (%)	457 (6.81%)	299 (4.46%)	244 (3.64%)
Crude HR (95% CI)	─	1.232 (1.040–1.459)*	1
Adjusted HR (95% CI)	─	1.129 (0.947–1.346)	1
Crude HR (95% CI)	1.544 (1.335–1.786)***	1	─
Adjusted HR (95% CI)	1.486 (1.283–1.721)***	1	─

†CI: Confidence interval.

† ***p < 0.001,

*p < 0.05.

†HRs were adjusted for age, gender, hypertension, depression, stroke, hyperlipidemia, atherosclerosis, alcohol abuse, anti-Parkinson, antithrombotics, antipsychotics, benzodiazepines, antidepressants, antiarrhythmics, statins.

**Table 3:** Risk of dementia between only PPIs, only H2RAs, and study group II.

Results	Study group I		
	Only PPIs	Only H2RAs	Study Group II
	*n* = 494	*n* = 1679	*n* = 6711
Dementia (%)	48 (9.72%)	128 (7.62%)	299 (4.46%)
Crude HR (95% CI)	2.267 (1.672–3.075)***	1.737 (1.412–2.136)***	1
Adjusted HR (95% CI)	1.836 (1.345–2.507)***	1.359 (1.102–1.676)**	1
Crude HR (95% CI)	1.305 (0.937–1.818)	1	─
Adjusted HR (95% CI)	1.462 (1.038–2.057)*	1	─

†CI: Confidence interval.

† ***p < 0.001,

**p < 0.01,

*p < 0.05.

†HRs were adjusted for age, gender, hypertension, stroke, hyperlipidemia, atherosclerosis, alcohol abuse, depression, anti-Parkinson, antithrombotics, antipsychotics, benzodiazepines, antidepressants, antiarrhythmics, statins.

**Fig 2:**
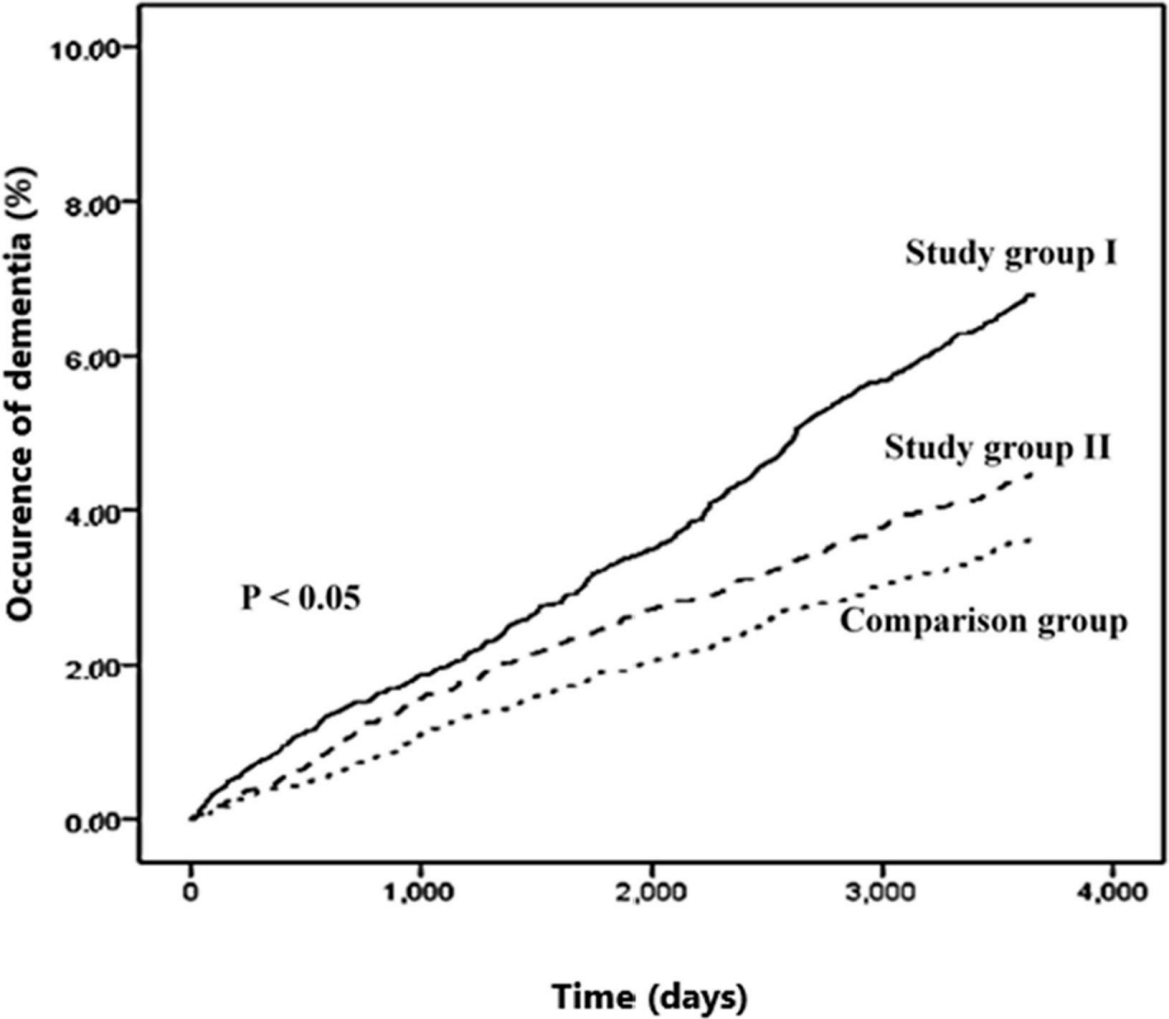
Kaplan–Meier curve of the cumulative occurrence of dementia among the three groups.

The Kaplan–Meier curve of the cumulative occurrence of dementia among the three groups revealed that patients with UGID receiving gastric acid–suppressive agents had the highest cumulative incidence of dementia (Fig 2).

## Discussion

This is the first retrospective cohort study to evaluate the risk of both PPIs and H2RAs on dementia in Asian patients with UGID. Our results demonstrate that the use of gastric acid–suppressive agents, both PPIs and H2RAs, increases the risk of dementia in patients with UGID and the use of PPIs involves significantly greater risk than the use of H2RAs.

Several mechanisms are related to our finding. For PPIs, first, the use of PPIs is associated with a higher β-amyloid (Aβ) level in the brain by affecting the enzymes β- and γ-secretases [6,7], which are involved in the pathogenesis of Alzheimer disease, the most common type of dementia. Second, PPIs may reduce the degradation of Aβ by lysosomes in microglia by inhibiting V-ATPases, thus increasing Aβ levels [8,9]. For H2RA, the anticholinergic effect caused by certain H2RAs, such as ranitidine and cimetidine, may lead to delirium and other cognitive deficits [10–12]. For both PPIs and H2RA, two kinds of medications could result in vitamin B12 deficiency, which is associated with cognitive decline [13]. In addition, gut microbiota play a major role in bidirectional communication between the gastrointestinal tract and central nervous system—called the microbiota–gut–brain axis. Thus, alteration of the composition of gut microbiota caused by the disruption of the low-pH environment by gastric acid–suppressive agents may negatively affect central nervous system function [14,15].

Our result can be supported by Makunts T, et al. study [16], which use data base from FDA adverse event reporting system, showed that PPI use significantly increase the memory impairment risk compared to H2RA use. However, in Makunts’s study due to the limitations stem from the occasionally missing demographic variables, treatment duration and lack of medical record data, not only the baseline characteristics including age and sex were significantly different between two groups, but also the confounding factors such as concurrent medications and comorbidities were not adjusted, which may cause bias in statistical analysis and results. In addition, the indication bias caused by PPI and H2RA were appeared, thus the memory impairment caused by initial disease state cannot be ruled out.

Our study has several noteworthy strengths. First, it is the first cohort study to evaluate the impact of gastric acid–suppressive agents, both PPIs and H2RAs, on dementia risk in Asian patients based on a large sample. Individuals over the age of 20 who met the selection criteria were included, and thus, the results are not limited to the elderly population. Second, excluding those receiving gastric acid–suppressive agents fewer than 90 days within 365 days after being first administered an agent may have ensured that all drug users had used a PPI or H2RA drug for a sufficient period of time (rather than accidentally), making the relationship between gastric acid–suppressive agents and dementia clearer. Third, a 10-year follow-up is in line with the pathogenesis of dementia and may have reduced the bias caused by censoring. Fourth, by using Taiwan’s LHID, we could both perform the analysis under real-life circumstances and minimize selection and recall bias. Fifth, we have included UGID in the analysis to prevent indication bias from affecting the results. Finally, we adjusted for numerous potential factors that influence the occurrence of dementia, including age, sex, alcohol abuse, tobacco dependence, comorbidities, and drug use.

Nevertheless, limitations remain. One is that factors such as educational attainment and the ApoE4 allele could not be estimated because the data source lacks sociodemographic and genetic records. Another is that information regarding patients’ adherence to gastric acid–suppressive agents could not be evaluated; nevertheless, the definition of PPI and/or H2RA use was the use of PPIs and/or H2RAs for at least 90 days within 365 days after the first administration, and thus, these patients all exhibited long-term use of PPIs and/or H2RAs to treat UGID. The cause–consequence relationship between gastric acid–suppressive agents and the risk of dementia was greatly increased. In conclusion, we conducted a retrospective cohort study with 10-year follow-up using data derived from the LHID and confirmed that using gastric acid–suppressive agents, including PPIs and H2RAs, is associated with an increased risk of dementia. Thus, restrictive and appropriate use of PPIs and H2RAs may help prevent the occurrence of dementia, and patients should be educated on medication adherence to shorten the treatment period during which they take gastric acid secretion inhibitors. The possible underlying causal biological mechanism must be explored in future studies. This finding should be further examined with a larger population and longer follow-up period.

## Conclusions

Our results demonstrate an increased risk of dementia in patients with UGID receiving gastric acid–suppressive agents, including PPIs and H2RAs, and the use of PPIs was associated with a significantly greater risk than H2RA use.
